# ﻿Big diversity in a small hotspot: two new species of Leptophlebiidae (Insecta, Ephemeroptera) from New Caledonia

**DOI:** 10.3897/zookeys.1143.96148

**Published:** 2023-01-25

**Authors:** Ľuboš Hrivniak, Michel Sartori, Pavel Sroka, Jindřiška Bojková

**Affiliations:** 1 Biology Centre of the Czech Academy of Sciences, Institute of Entomology, Branišovská 31, 37005 České Budějovice, Czech Republic Biology Centre of the Czech Academy of Sciences, Institute of Entomology České Budějovice Czech Republic; 2 Musée cantonal de zoologie, Palais de Rumine, Place de la Riponne 6, 1014 Lausanne, Switzerland Museum of Zoology Lausanne Switzerland; 3 Department of Ecology and Evolution, Biophore, University of Lausanne, 1015 Lausanne, Switzerland University of Lausanne Lausanne Switzerland; 4 Department of Botany and Zoology, Faculty of Science, Masaryk University, Kotlářská 2, 61137 Brno, Czech Republic Masaryk University Brno Czech Republic

**Keywords:** Atalophlebiinae, Australasia, barcoding, mayflies, taxonomy

## Abstract

Two new species from Grande Terre Island, New Caledonia, namely *Fasciamiruspetersorum***sp. nov.** and *Simulacalarara***sp. nov.** are described based on larval morphology and molecular data (COI sequences). *Fasciamiruspetersorum***sp. nov.** is distributed in the southern part of the island and is characterised by a reduced third segment of the labial palps and all abdominal gills divided from the base. The species inhabits slow-flowing aquatic habitats with fine-grained substrate in forest brooks. *Simulacalarara***sp. nov.** is known from a single locality in the northern part of the island and is characterised by narrow and distinctly elongated abdominal gills 1–7. It was collected from fine substrates behind stones in riffles with slightly turbulent flow. Both species were recorded only in areas with ultramafic bedrock.

## ﻿Introduction

**Figure 1.**
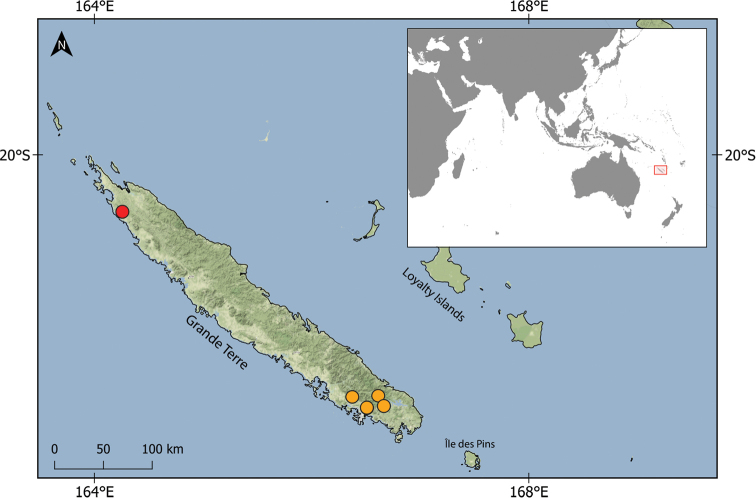
Location of New Caledonia and distribution of the two new species in Grande Terre, *Fasciamiruspetersorum* sp. nov. – orange circles and *Simulacalarara* sp. nov. – red circle.

New Caledonia, a Melanesian archipelago located about 1700 km northeast of New Zealand, represents one of the smallest biodiversity hotspots in the world (Grandcolas 2017). Its largest island, Grande Terre (Fig. 1), is characterised by a highly diverse landscape due to varied geological, topographic and climatic conditions, which is crucial for terrestrial and freshwater biodiversity (Murienne 2009). Freshwater habitats of Grande Terre mostly consist of small fast-flowing streams and brooks hosting several species-rich groups of aquatic insects, mainly aquatic Coleoptera, Trichoptera, Odonata, and Ephemeroptera (Peters 2001; Jäch and Balke 2010; Grand et al. 2014; Johanson and Wells 2019). Of these, Ephemeroptera exhibit an exceptional level of endemism, with 19 genera endemic to New Caledonia, compared to, for example, only five endemic genera known in species-rich Trichoptera (Johanson and Wells 2019) or no endemic genus in aquatic Heteroptera and Odonata (Damgaard and Zettel 2014; Grand et al. 2014). Most aquatic insects endemic to New Caledonia belong to genera distributed throughout Australasia.

The research on the mayflies in New Caledonia has been led by William L. Peters and Janice G. Peters, who published a series of eight articles focusing on taxonomy and systematics (Peters et al. 1978; Peters and Peters 1980, 1981a, b, 2000; Peters et al. 1990, 1994; Peters and Mary 2016). New Caledonian mayflies belong to a single family Leptophlebiidae and subfamily Atalophlebiinae (Peters 2001). All globally distributed (and relatively vagile) taxa often inhabiting remote islands, such as Baetidae and Caenidae (Jacobus et al. 2019), are absent there. The only exceptions were two adult specimens of the baetid genus *Pseudocloeon* (?) found in Rivière Bleue (Peters et al. 1978). However, no further specimens of Baetidae have been collected from this area since then, and the occurrence of this family in New Caledonia is therefore doubtful.

Leptophlebiidae of New Caledonia have diversified into the following 19 endemic genera with a total species richness of 46 species/subspecies (Mary 2017): *Lepeorus* Peters, Peters & Edmunds, 1978 (three species and two subspecies), *Lepegenia* Peters, Peters & Edmunds, 1978 (one species), *Celiphlebia* Peters & Peters, 1980 (two species), *Tindea* Peters & Peters, 1980 (one species), *Poya* Peters & Peters, 1980 (one species), *Peloracantha* Peters & Peters, 1980 (one species), *Coula* Peters & Peters, 1980 (one species), *Notachalcus* Peters & Peters, 1981 (one species), *Ounia* Peters & Peters, 1981 (one species), *Papposa* Peters & Peters, 1981 (one species), *Kariona* Peters & Peters, 1981 (one species), *Fasciamirus* Peters, Peters & Edmunds, 1990 (one species), *Simulacala* Peters, Peters & Edmunds, 1990 (three species), *Kouma* Peters, Peters & Edmunds, 1990 (four species), *Tenagophila* Peters, Peters & Edmunds, 1994, (two species), *Oumas* Peters & Peters, 2000 (one species), *Paraluma* Peters & Peters, 2000 (six species), *Amoa* Peters & Peters, 2000 (five species and one subspecies), *Neampia* Peters & Mary, 2016 (subgenera: *Neampia* s.s., three species; *Goa*, one species; *Adonela*, two species).

In addition to the remarkable number of endemic genera, New Caledonian mayflies are exceptional because they are able to occupy different freshwater microhabitats due to great eco-morphological diversification. Here we describe two new species from morphologically related genera, *Fasciamirus* and *Simulacala*, the larvae of which inhabit small stones in quiet sections of streams and burrow in fine gravel in streams and rivers, respectively (Peters et al. 1990).

We combine both morphological and molecular data (COI sequences) to delimit them and provide larval morphological differential diagnostics. We also provide basic information on their habitat preferences and distribution. This study provides the first molecular data on the genera *Fasciamirus* and *Simulacala* endemic to New Caledonia. The definition of these two very similar genera and the generic attribution of both new species are also discussed.

## ﻿Material and methods

The material of mayfly larvae used for this study was collected by J. Bojková and Ľ. Hrivniak in January/February 2022. All specimens were preserved in 96% EtOH and are deposited in the collections of the Biology Centre of the Czech Academy of Sciences, Institute of Entomology, České Budějovice, Czech Republic (**IECA**) and the Museum of Zoology, Lausanne, Switzerland (**MZL**). Type material from the MZL was used for the morphological comparison between larvae of new species and other species from the genera *Simulacala* and *Fasciamirus*.

### ﻿Molecular data

Total genomic DNA of 25 specimens was extracted from larval legs using the DEP-25 DNA Extraction Kit (TopBio, Prague, Czech Republic) according to the manufacturer’s protocol. Polymerase Chain Reaction (PCR) amplification of mitochondrial cytochrome oxidase subunit I (COI) was sequenced according to Hrivniak et al. (2017). Sequencing was carried out in SeqMe (Dobříš, Czech Republic).

DNA from 10 specimens (with the Swiss Global Biodiversity Information Facility codes; GBIFCH in Table 1) was extracted according to the protocol by Vuataz et al. (2011). COI was amplified using HCO2198 and LCO1490 primers (Folmer et al. 1994). PCR was conducted in a volume of 25 μl, consisting of 5 μl of template DNA, 1.3 μl (10 μM) of each primer, 0.2 μl (25 mM) of dNTP solution (Promega), 5 μl of 5X buffer (Promega) containing 7.5 mM of MgCl_2_, 2.5 μl (25 mM) of MgCl_2_, 1 U of Taq polymerase (Promega), and 9.7 μl of sterile ddH_2_O. Optimized PCR conditions included initial denaturation at 95 °C for 5 min, 40 cycles of denaturation at 95 °C for 30 s, annealing at 50 °C for 30 s, and extension at 72 °C for 40 s, with final extension at 72 °C for 7 min. Sequencing was carried out in Microsynth (Balgach, Switzerland). Sequences were assembled in Geneious ver. 7.0.6 (http://www.geneious.com) and aligned in the same software using the Mafft ver. 7.017 plugin (Katoh et al. 2002) with default settings. The sequences obtained are deposited in GenBank with the accession numbers listed in Table 1.

**Table 1.** Specimens used for sequencing of COI and GenBank codes.

Species	Specimen code	GenBank
*Fasciamirusrae*	FR1	OP970180
*Fasciamirusrae*	FR2	OP970181
*Fasciamirusrae*	FR3	OP970182
*Fasciamirusrae*	FR4	OP970196
*Fasciamirusrae*	FR5	OP970197
*Fasciamirusrae*	FR6	OP970198
*Fasciamirusrae*	GBIFCH00970348	OP970186
*Fasciamiruspetersorum* sp. nov.	FN1	OP970189
*Fasciamiruspetersorum* sp. nov.	FN2	OP970190
*Fasciamiruspetersorum* sp. nov.	FN3	OP970191
*Fasciamiruspetersorum* sp. nov.	FN4	OP970192
*Fasciamiruspetersorum* sp. nov.	FN5	OP970193
*Fasciamiruspetersorum* sp. nov.	FN6	OP970194
*Fasciamiruspetersorum* sp. nov.	FN7	OP970195
*Simulacalarara* sp. nov.	S1	OP970199
*Simulacalarara* sp. nov.	S2	OP970200
*Simulacalarara* sp. nov.	GBIFCH01121825	OP970213
*Simulacalarara* sp. nov.	GBIFCH01121826	OP970214
*Simulacalanotialis*	SN1	OP970184
*Simulacalanotialis*	SN3	OP970185
*Simulacalanotialis*	SN4	OP970206
*Simulacalanotialis*	SN5	OP970207
*Simulacalanotialis*	GBIFCH01121829	OP970212
*Simulacalanotialis*	GBIFCH01121836	OP970211
*Simulacalanotialis*	GBIFCH00970320	OP970188
*Simulacalamassula*	SM2	OP970183
*Simulacalamassula*	SM3	OP970201
*Simulacalamassula*	SM4	OP970202
*Simulacalamassula*	GBIFCH01121828	OP970208
*Simulacalamassula*	GBIFCH00970322	OP970187
*Simulacalamilleti*	SMI1	OP970203
*Simulacalamilleti*	SMI2	OP970204
*Simulacalamilleti*	SMI3	OP970205
*Simulacalamilleti*	GBIFCH01121827	OP970209
*Simulacalamilleti*	GBIFCH01121835	OP970210

### ﻿Molecular species delimitation

Molecular delimitation of species was performed using the General Mixed Yule Coalescent model (GMYC, Pons et al. 2006) and the Assemble Species by Automatic Partitioning (ASAP; Puillandre et al. 2021). GMYC was performed using the SPLITS package for R. An ultrametric COI gene tree was reconstructed using BEAST 2 (Bouckaert et al. 2014) with settings described in Hrivniak et al. (2020). Two analyses were running on CIPRES Science Gateway 3.3 (Miller et al. 2010) for 500 million generations sampled every 50 000 generations. Convergence and effective sample size (ESS > 200) were verified using Tracer ver. 1.6. The first 10% of trees from each run were discarded as burn-in. Files from both independent runs were combined using LogCombiner ver. 1.8.4. The maximum clade credibility tree was constructed using TreeAnnotator ver. 1.8.4 with default settings. Nodes on the tree with posterior probability (PP) below 0.95 were collapsed. ASAP analysis was run on the online graphical web interface available at https://bioinfo.mnhn.fr/abi/public/asap/. The input dataset for ASAP comprised sequences aligned in a fasta file. The simple pairwise genetic distances were selected and other settings were default. Inter- and intraspecific pairwise genetic distances were calculated in MEGA ver. 7 (Kumar et al. 2016).

### ﻿Morphological examination

Parts of specimens (larvae) were mounted on microscopic slides using HydroMatrix (MicroTech Lab, Graz, Austria) mounting medium. In order to remove muscle tissue for examination of cuticular structures, specimens were left overnight in a 10% NaOH solution before mounting on slides. Drawings were made using Olympus SZX7 stereomicroscope and Olympus BX41 compound microscope, both equipped with a drawing tube. Photographs of larvae were taken with Canon EOS 6D camera and processed using Adobe Photoshop Lightroom (http://www.adobe.com) and Helicon Focus ver. 5.3 (http://www.heliconsoft.com). Photos of mouthparts mounted on microscopic slides were taken with a Keyence VHX-750. All photographs were subsequently enhanced using Adobe Photoshop CS5. Larval morphological diagnostic characters and terminology for taxonomic descriptions were adopted from Peters et al. (1990) and Mary (2017).

## ﻿Results and discussion

### ﻿Molecular species delimitation

The final COI alignment contained 623 base pairs and 207 parsimony informative positions.

**Figure 2.**
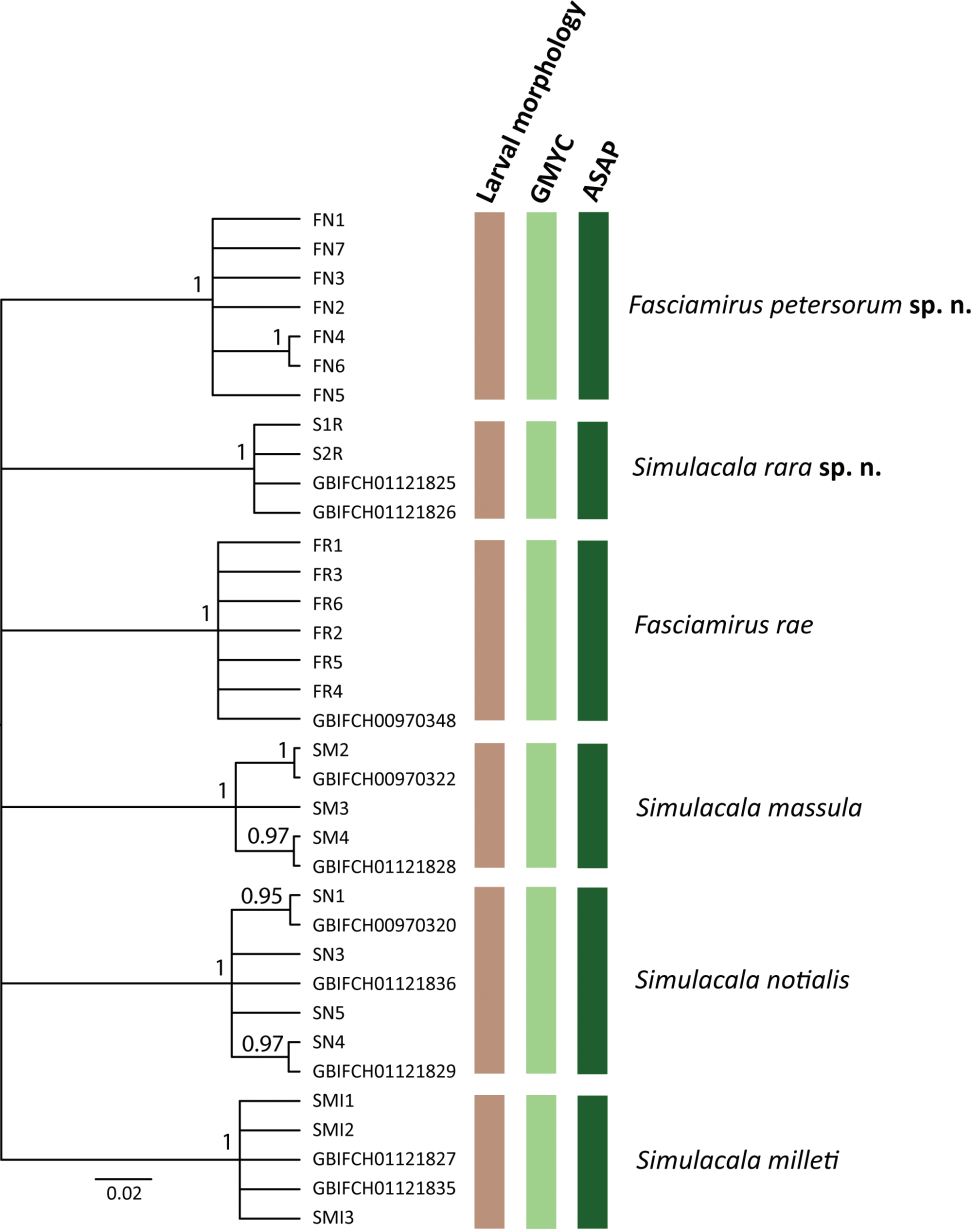
: Maximum clade credibility tree of the COI dataset generated in BEAST2 and species delimitation based on the comparative morphology of larvae, GMYC and ASAP. Numbers near nodes indicate posterior probability from the BEAST2 analysis.

Both species delimitation methods, GMYC and ASAP, identified six species (Fig. 2) within the COI dataset. All morphologically defined species used in the study, including the two putative new species, were recognized as distinct species and all species formed fully supported monophyletic clades (PP = 1, Fig. 2). Pairwise intraspecific genetic distances in *Fasciamiruspetersorum* sp. nov. reached 2.7% and in *Simulacalarara* sp. nov. reached 0.6%. Pairwise genetic distances between *F.petersorum* sp. nov. and *F.rae* ranged between 13.7 and 15.7%. Interspecific distances between *Simulacalarara* sp. nov. and its congeners ranged between 18.9–20.5%.

### ﻿Taxonomy

#### ﻿ Fasciamirus petersorum

Taxon classificationAnimaliaEphemeropteraLeptophlebiidae

Hrivniak & Bojková sp. nov.

C11949A5-C54E-5B6A-AA96-083CD15E848C

https://zoobank.org/F1FC1C40-C49C-45CC-BF8D-E7C9CE961991

##### Material examined.

***Holotype*.** Female larva, New Caledonia; Les Koghis, headwaters of Ouanéoué (Loc. 59/2022), -22.1755278, 166.5094167, 510 m a.s.l.; 22.01.2022; leg. J. Bojková, Ľ. Hrivniak. Deposited in MZL. ***Paratypes*.** 33 larvae, same data as holotype. Deposited in MZL. 12 larvae, New Caledonia; left tributary of Rivière Bleu, Cornes du diable (Loc. 63/2022), -22.0691111, 166.6130000, 350 m a.s.l.; 22.01.2022; leg. J. Bojková, Ľ. Hrivniak (9 larvae deposited in MZL, 3 larvae mounted on slides and deposited in IECA—DNA extracted). 4 larvae, New Caledonia; Païta, Carignan above villages of Païta (Loc. 56/2022), -22.0785278, 166.3746944, 170 m a.s.l.; 20.01.2022; leg. J. Bojková, Ľ. Hrivniak (2 larvae deposited in MZL; 2 larvae mounted on slides and deposited in IECA—DNA extracted). 4 larvae (1 mounted on slide—DNA extracted), New Caledonia; Route de la Rivière Blanche, unnamed brook, (Loc. 46/2022), -22.1586667, 166.6652222, 175 m a.s.l.; 25.01.2022; leg. J. Bojková, Ľ. Hrivniak. Deposited in IECA. 2 larvae (1 mounted on slide—DNA extracted), New Caledonia; Païta, small brook near the river (Loc. 57/2022), -22.0790556, 166.3742222, 170 m a.s.l.; 20.01.2022; leg. J. Bojková, Ľ. Hrivniak. Deposited in IECA.

##### Description of larva.

Body length of female late-instar larvae 8.0 mm, male 6.0–7.0 mm. Body covered with sparse hair-like setae.

**Figure 3.**
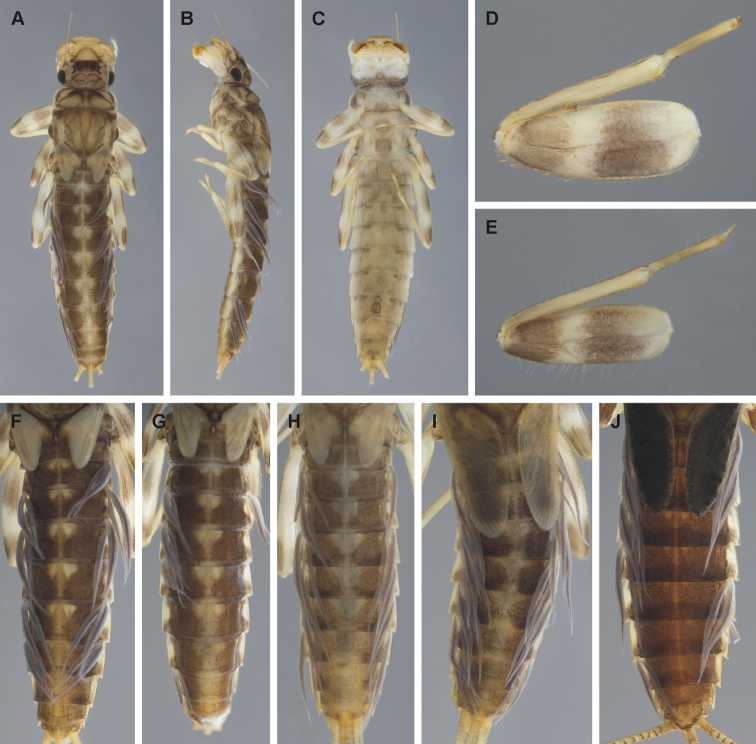
*Fasciamiruspetersorum* sp. nov., larva: **A** habitus dorsal **B** habitus lateral **C** habitus ventral **D, E** foreleg **F–J** abdomen.

***Head*.** Prognathous, antennae more than 2× longer than head. Color light brown with dark brown markings between ocelli and antennae as in Fig. 3A.

**Figure 4.**
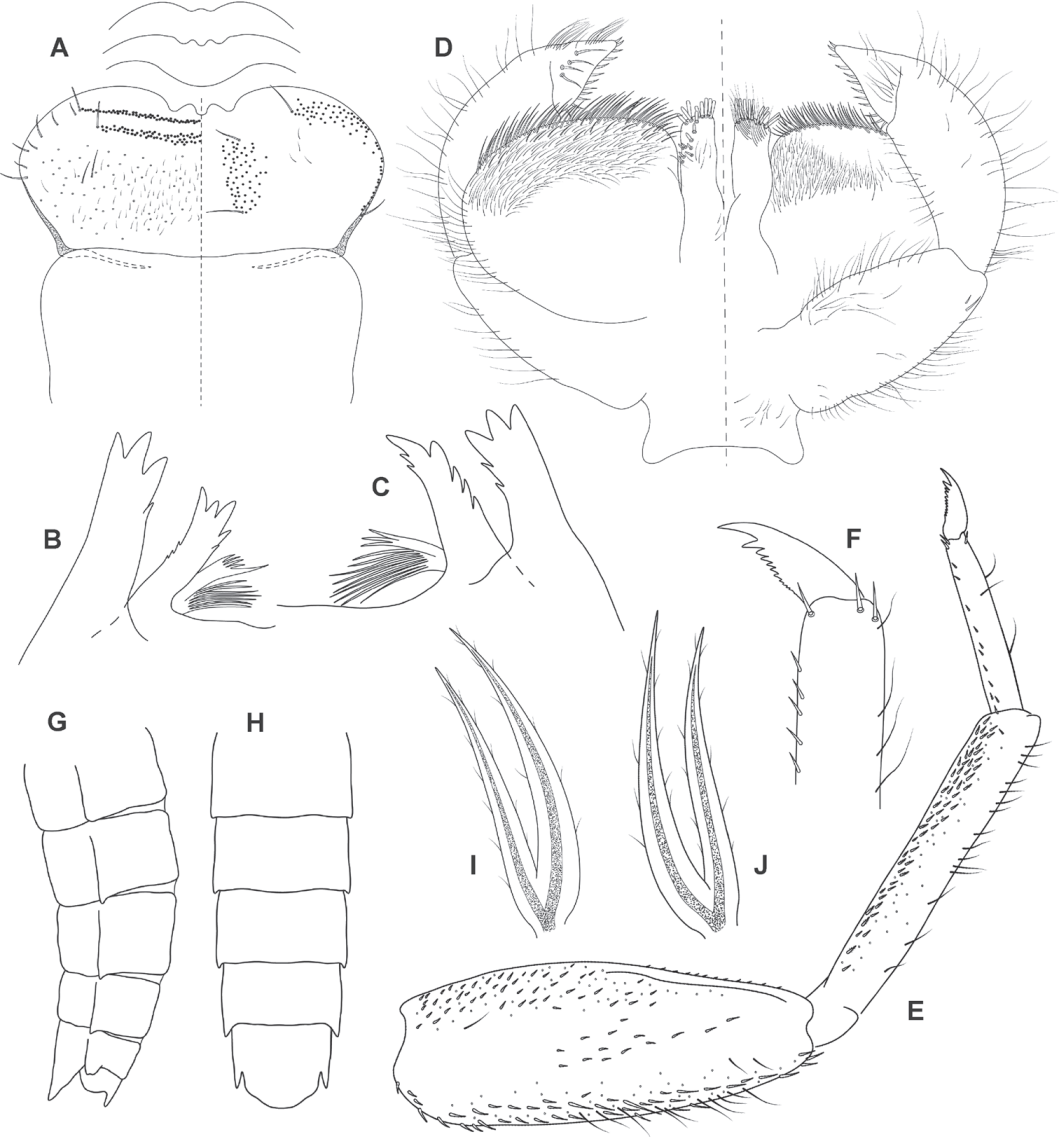
*Fasciamiruspetersorum* sp. nov., larva: **A** labrum (left half dorsal, right half ventral), clypeus, and variability of anterior margin of labrum **B** incisors and prostheca of left mandible **C** incisors and prosteca of right mandible **D** labium (left half dorsal, right half ventral) **E** foreleg **F** tarsal claw of foreleg **G** abdomen in lateral view (segments V–X) **H** abdomen in ventral view (segments V–IX **I** gill I **J** gill IV.

***Mouthparts*.** Labrum and clypeus shape as in Fig. 4A. Labrum twice wider than long; clypeus of similar proportions or slightly narrower. Dorsal surface of labrum with scattered hair-like setae and two irregular rows of bristle-like setae along anterior margin (Fig. 4A, left half; setae marked with black dots). Anterior margin deeply cleft medially with 2 large denticles, sometimes reduced or absent (Fig. 4A). Ventral surface with long hair-like setae submedially and anterolaterally (Fig. 4A, right half; setae marked with black dots). Hypopharynx shape as in Fig. 6A. Lingua with well-developed lateral processes and anterior margin deeply cleft. Superlingua extended laterally with bristle-like setae along dorsal margin, lateral margins rounded (Fig. 6A). Both mandibles with two incisor groups, separated from base and equipped with denticles (Fig. 4B, C). Prostheca of right and left mandible similar, divided into two branches, one comb-like, second with long filaments apically (Fig. 4B, C). Maxillae shape and setation as on Fig. 6B. Maxillary palps three-segmented, first and second segment of approximately same length, length of third segment 0.61–0.72 of second segment. Third segment triangular, broad at base. Shape of glossae and paraglossae as in Fig. 4D. Labial palps three-segmented, first and second segment of approximately same length, length of third segment 0.35–0.41 of second segment. Third segment triangular, broad at base with spine-like setae on inner and dorsal margins (Fig. 4D, left half).

***Thorax*.** Light brown with dark brown markings dorsally. Ganglia darkened. (Fig. 3A–C).

***Legs*.** Femora with dark brown macula near apex and base as in Fig. 3D, E. Tibiae and tarsi yellowish brown. Maximum width of tibiae 1.62–2.00× maximum width of tarsi. Tibiae oval in cross-section. Inner margins of femora indented in apical half. Claws with denticles apically progressively larger (Fig. 4F).

***Abdomen*.** Terga dark brown with pale markings as in Fig. 3F–J. Shape of pale markings vary from triangular (Fig. 3G–I) to longitudinal stripe with pair of lateral spots (Fig. 3F, J). Markings most expressed on terga II–VI. Sterna I–IX with medio-lateral brown maculae (Fig. 3C) and darkened ganglia. Posterolateral spines on abdominal segments (V)VI–IX (Fig. 4G, H). Spine on segment IX apically indented. Sternum IX widely rounded posteriorly (Fig. 4H).

***Gills*.** On abdominal segments I–VII. Shape of all gills alike, lanceolate, elongated, and smoothly tapered to apex (Fig. 4I, J). All gills divided from near base. Lamellae grey, tracheae blackish.

***Caudal filaments*.** Yellowish brown, terminal filament little longer that cerci. Length of cerci approximately 1.4× body length.

##### Subimago, imago, and egg.

Unknown.

##### Etymology.

The species is named in honour of Janice G. Peters and William L. Peters, who discovered and described an amazing variety of New Caledonian mayflies. Plural.

##### Generic attribution.

The larva of the genus *Fasciamirus* was defined by Peters et al. (1990) based on the following morphological characters: i) inner margin of third segment of labial palps has thick heavy spines; ii) glossae of labium are straight; iii) maximum width of tibiae is 2 times maximum width of tarsi; (iv) abdominal gills I differ from gills II–VII; gill I is long, slender, usually without fork while gills II–VII are forked and each portion is long and smoothly tapered to apex; distance from base to fork of gills II–V exceeds length of ensuing segment; and (v) denticles on claws are progressively larger apically, except the apical denticle is a little larger. *Fasciamirus* is also characterized by specific coloration pattern of femora in larval stages and adults consisting of dark brown macula near apex and base (Peters et al. 1990: fig. 119).

It should be noted that the genus *Fasciamirus* was described based only on a single species, *F.rae* Peters, Peters & Edmunds, 1990. Therefore, larger variability in some characters can be expected when other congeneric species are described. The new species *F.petersorum* sp. nov. possesses most of the morphological characters of the genus as defined by Peters at al. (1990). The exception are gills that are all alike and divided near the base. We have also found variability in the width of tibiae in respect to the width of tarsi. While Peters at al. (1990) defined the width of tibiae as 2× width of tarsi, we found that the width of tibiae varies between 1.62–2.00× maximum width of tarsi. Despite these incongruences, most generic characters defining *Fasciamirus* are shared in *F.petersorum* sp. nov. Therefore, we are confident that the new species belongs to the genus *Fasciamirus* and the differences from *F.rae* represent intrageneric variability.

Finally, the attribution of the new species to the genus *Fasciamirus* is based on the synthesis of larval morphological characters given by Peters et al. (1990) and the identification key to the genera of New Caledonian Leptophlebiidae by Mary (2017) as follows: i) femora with distinct coloration pattern consisting of dark brown maculae near the apex and base (Fig. 3D, E); ii) gills lanceolate, narrow (width of both branches less than 1/3 of the length), elongated and gently tapering to the apex (Fig. 4I, J); iii) third segment of labial palps broad, triangular, with spine-like setae on inner margins (Fig. 4D); iv) glossae of labium are straight (Fig. 4D); and v) denticles on claws are progressively larger apically, except the apical denticle is a little larger (Fig. 4F). Additionally, we add the narrow triangular shape of the third segment of maxillary palps (Peters et al. 1990: fig. 104; Fig. 6B) among generic diagnostic characters of *Fasciamirus*.

##### Larval morphological diagnostics.

*Fasciamiruspetersorum* sp. nov. can be distinguished by a combination of the following characters: i) length of the third segment of labial palps reaching 0.35–0.41 times that of the second segment (Fig. 4D,); ii) gills on abdominal segments I–VII similar and divided from the base (Fig. 4I, J); and iii) anterior margin of labrum with two large denticles medially (sometimes reduced, Fig. 4A).

##### Differential diagnosis.

The species *F.petersorum* sp. nov. is the second species described in the genus *Fasciamirus*. It can be distinguished from *F.rae* by the following characters: i) gills I–VII are similar and all divided from the base (Fig. 4I, J), in contrast to *F.rae*, in which the first pair of gills is usually undivided, forming a single filament, and gills II–VI are divided at ¼ from the base (Peters et al. 1990: figs 121, 122); ii) the third segment of labial palps is reduced, its length reaching only to about ¼ of the second segment (Fig. 4D) [In *F.rae*, the third segment of labial palps reaches up to 3/5 of the second segment length and is thus only slightly shorter than the second segment (Peters et al. 1990: fig. 117)]; and iii) the anteromedian margin of labrum has two large denticles that are sometimes reduced (Fig. 4A), in contrast to *F.rae* with 5 (rarely 6) denticles (Peters et al. 1990: fig. 100). The reduction of denticulation on the anteromedian margin of labrum in *F.petersorum* sp. nov. was found in genetically similar specimens and represents intraspecific variation.

##### Distribution and habitat preferences.

The species is distributed in the southern province of Grande Terre (Fig. 1) on ultramafic bedrock. It was found only in clear brooks flowing in pristine (or near-natural) forests, in the vicinity of Mounts Koghis near Dumbéa, Mont Mou near Païta, and Rivière Bleu. Despite low altitude (170–510 m a.s.l.), the brooks have a mountainous character and are relatively cold (19–22 °C in summer; other streams were usually 25–30 °C at that time). They included very small (less than one metre wide) or small (mean width 2–6 m) cascading brooks with prevailing turbulent flow and stony substrate (Fig. 7). However, larvae were collected in slow-flowing microhabitats with sandy and fine gravel substrate in pools.

#### ﻿ Simulacala rara

Taxon classificationAnimaliaEphemeropteraLeptophlebiidae

Hrivniak & Bojková sp. nov.

0573CA05-0E89-5338-9878-ACA919338F96

https://zoobank.org/5A2F303F-9F02-44C7-A511-AC1BAE447ABB

##### Material examined.

***Holotype*.** Female larva, New Caledonia; Chagrin, Fridoline River. (Loc. 98/2022), -20.4902778, 164.2572222, 70 m a.s.l.; 03.02.2022; leg. J. Bojková, Ľ. Hrivniak. Deposited in MZL. ***Paratypes*.** 2 larvae (1 larva mounted on slide and deposited in IECA, 1 larva deposited in MZL—DNA extracted from both), same data as holotype.

##### Other material examined

**(used for DNA extraction; cuticular skin preserved and deposited in MZL).** 2 larvae (GBIFCH01121825 and GBIFCH01121826), same data as holotype.

##### Description of larva.

Body length of middle-instar larvae 3.0–3.5 mm. Size of late-female and male instar larvae unknown. General coloration of body yellowish with dark brown markings. Body covered by sparse hair-like setae.

**Figure 5.**
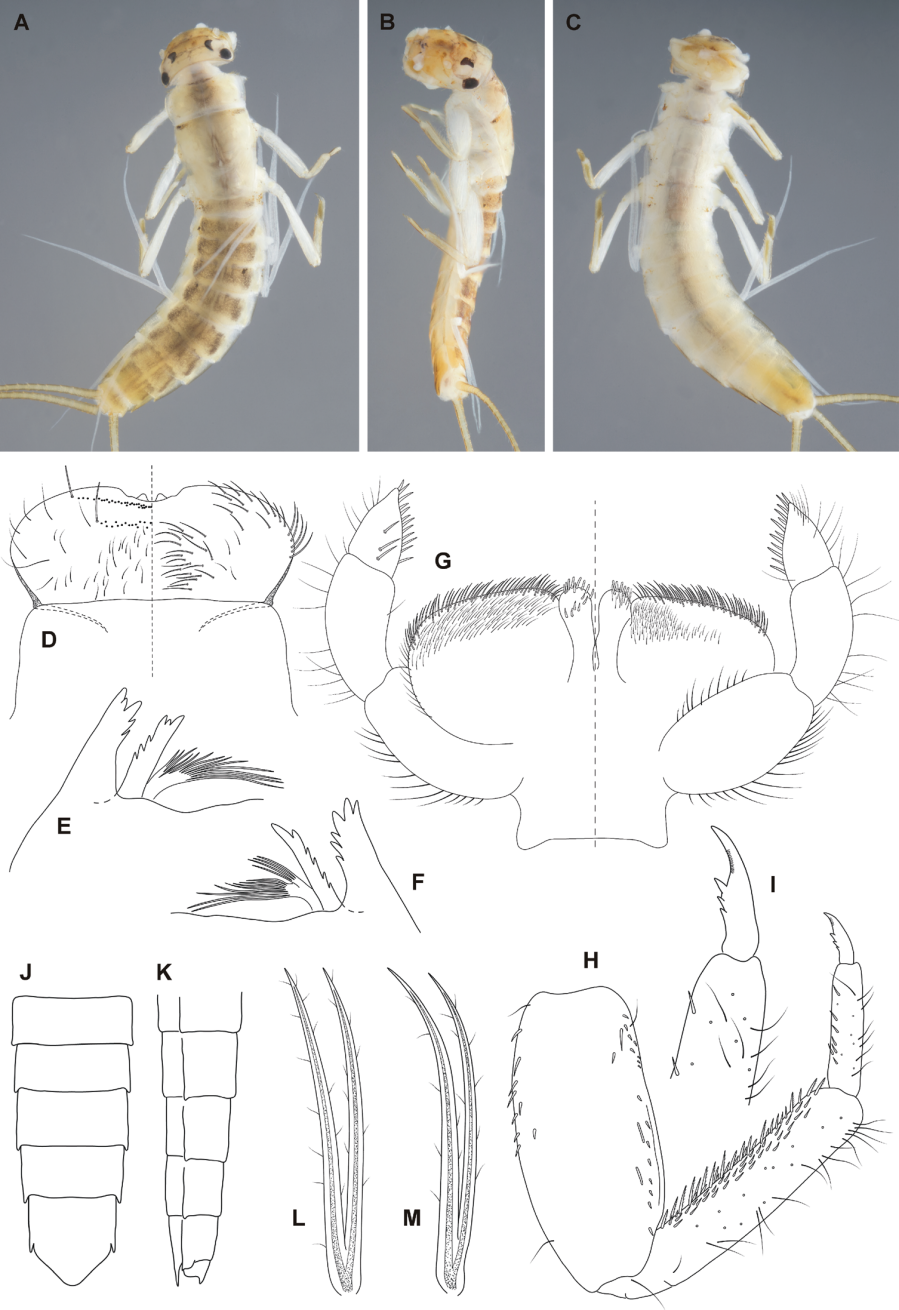
*Simulacalarara* sp. nov., larva: **A** habitus dorsal **B** habitus lateral **C** habitus ventral **D** labrum (left half dorsal, right half ventral) and clypeus **E** incisors and prostheca of left mandible **F** incisors and prostheca of right mandible **G** labium (left half dorsal, right half ventral) **H** foreleg **I** tarsal claw of foreleg **J** abdomen in ventral view (segments V–IX) **K** abdomen in lateral view (segments V–X) **L** gill V **M** gill I.

***Head*.** Prognathous, antennae more than 2× longer than head. Coloration yellowish with dark brown markings as in Fig. 5B.

**Figure 6.**
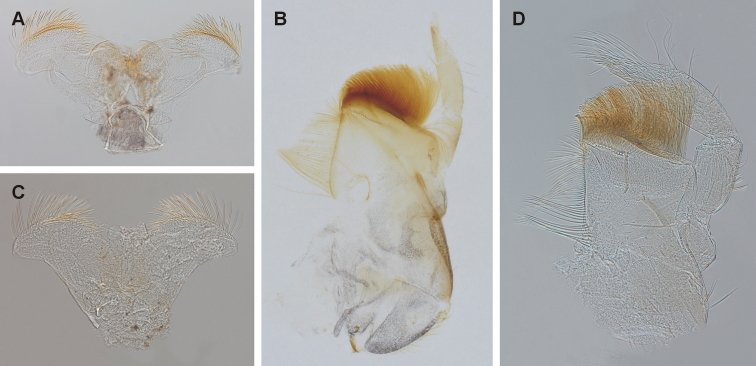
**A** hypopharynx of *Fasciamiruspetersorum* sp. nov. **B** maxilla of *F.petersorum* sp. nov. **C** hypopharynx of *Simulacalarara* sp. nov. **D** maxilla of *S.rara* sp. nov.

***Mouthparts*.** Shape of labrum and clypeus as in Fig. 5D. Labrum ca. 2.3× wider than long. Length and width of labrum approximately same as length and width of clypeus (Fig. 5D). Dorsal surface of labrum with scattered hair-like setae and two irregular rows of bristle-like setae along anterior margin (Fig. 5D, left half; setae marked with dots). Anterior margin medially deeply cleft, with two large denticles (Fig. 5D). Ventral surface with long hair-like setae submedially and anterolaterally (Fig. 5D, right half). Lingua with well-developed lateral processes and deeply cleft anterior margin. Superlinguae laterally extended with bristle-like setae along dorsal margins, lateral margins acute (Fig. 6C). Both mandibles with two incisor groups, separated from base and equipped with denticles (Fig. 5E, F). Prostheca of right and left mandible similar, indistinctly divided into two branches, each with long filaments apically. Shape and setation of maxillae as on Fig. 6D. Maxillary palps three-segmented, first and second segment of approximately same length, length of third segment 0.73–0.80× length of second segment. Third segment triangular and broad at base. Shape of glossae and paraglossae as in Fig. 5G. Labial palps three-segmented, length of second segment 0.82–0.95× length of first segment, length of third segment 0.61–0.68× length of second segment. Third segment triangular, broad at base, with spine-like setae on inner and dorsal margins (Fig. 5G).

***Thorax*.** Yellowish with dark brown markings dorsally (Fig. 5A–C).

***Legs*.** Yellowish, without apparent pattern (Fig. 5B, H). Maximum width of tibiae 1.41–1.60× width of tarsi. Tibiae oval in cross-section. Inner margins of femora indented in apical half. Claws with 3–5 large denticles (apically progressively longer); apical part of claws hooked with a row of small denticles as in Fig. 5I.

***Abdomen*.** Terga brownish with pale markings forming median stripe and pair of medio-lateral elongated maculae (Fig. 5A). Terga darkened sub-laterally. Sterna yellowish, without pattern (Fig. 5C). Postero-lateral spines on abdominal segments VI–IX (Fig. 5J, K). Spines on segment IX apically indented (Fig. 5K). Posterior portion of sternum IX triangular, rounded apically (Fig. 5J).

***Gills*.** On abdominal segments I–VII. Shape of all gills alike; all gills divided from near base. Each branch narrow and distinctly elongated (e.g. gill IV in middle-instar larvae reaches or exceeds end of abdomen), smoothly tapered to apex (Fig. 5A, L, M). Lamellae pale grey, tracheae greyish.

***Caudal filaments*.** Yellowish, terminal filament little longer than cerci. Cerci length approximately 1.2× body length.

##### Subimago, imago, and egg.

Unknown.

##### Etymology.

The species is named according to its rare occurrence in Grande Terre. Feminine.

##### Generic attribution.

The larva of the genus *Simulacala* was defined by Peters et al. (1990) based on the following morphological characters: i) inner margin of third segment of labial palps has thick heavy spines; ii) glossae of labium are straight; iii) denticles on claws are progressively larger apically, except apical denticle larger to much larger; iv) third segment of the maxillary palps is 3/4 to 4/5 length of second segment; and v) abdominal gills I–VII are deeply forked and 2 portions of lamellae overlap; each portion is long and smoothly to abruptly tapered to apex. Mary (2017) added two additional characters for the identification of the genus: i) anterior margin of labrum with 2 or 3 large denticles and ii) length of the distal segment of the labial palp approximately equal to its maximum width.

Almost all generic characters mentioned above correspond to *S.rara* sp. nov. suggesting its position in the genus *Simulacala*. The only exception is the length of the distal segment of the labial palps, reaching approximately 1.5 times its width. Nevertheless, other characteristics of labial palps, notably the presence of dense spines on the inner and dorsal margins correspond to the genus *Simulacala*.

The genus *Fasciamirus* is most closely related to *Simulacala* and their larvae possess similar morphological characters as indicated by Peters et al. (1990). They can be distinguished based on the presence of dark bands on femora of all legs in *Fasciamirus*, whereas these bands are absent in *Simulacala*; this applies to imagos, subimagos and larvae (Peters et al. 1990). According to our observation, this coloration pattern is visible already in younger larval instars of *Fasciamirus*. Femora of *S.rara* sp. nov. do not exhibit dark bands, suggesting an attribution to the genus *Simulacala*. Additionally, longer and wider triangular shape of the third segment of maxilary palp in *S.rara* sp. nov. is characteristic for the genus *Simulacala* (see Peters et al. 1990: fig. 110 and Fig. 6D). *Fasciamirus* possesses a rather narrower triangular and a shorter third segment of maxilary palps (see Peters et al. 1990: fig. 104 and Fig. 6B).

According to the larval morphological characters given by Peters et al. (1990) and Mary (2017), *S.rara* sp. nov. is attributable to the genus *Simulacala* based on following characters: i) third segment of labial palps broad, triangular, with spine-like setae on inner margins (Fig. 5G); ii) gills lanceolate, narrow (width of both branches less than 1/3 of length) and divided at base (Fig. 5L, M); iii) tibiae of all legs oval in cross-section; iv) denticles on claws are progressively larger apically with apical denticle larger to much larger (Fig. 5I); v) glossae of labium are straight (Fig. 5G); vi) segment 3 of the maxillary palps is 3/4 to 4/5 length of segment 2 (Fig. 6D); vii) femora of all legs without dark apical and distal maculae (Fig. 5B).

We do not rule out the possibility that *S.rara* sp. nov. may represent a separate monospecific genus, as the larval morphological convergences in New Caledonia are extreme and adults, bearing more informative systematic traits, are unknown. Nevertheless, the larvae of *S.rara* sp. nov. are morphologically most similar to the genus *Simulacala* as currently defined. Therefore, we describe this species therein until the large-scale revision of New Caledonian Atalophlebiinae is completed, based on larger number of mitochondrial and nuclear markers. This revision is currently in preparation.

##### Larval morphological diagnostics.

*Simulacalarara* sp. nov. can be distinguished by the combination of the following characters: i) all gills narrow, smoothly tapering to the apex, distinctly elongated and reaching or exceeding end of abdomen in middle-instar larvae (Fig. 5A, L, M); ii) femora and mesobasisternum without dark markings (Fig. 5B, H); iii) anteromedian margin of labrum with two large denticles (Fig. 5D).

##### Differential diagnosis.

Three other species of the genus *Simulacala* occur in Grande Terre: *Simulacalanotialis* Peters, Peters & Edmunds, 1990, *S.massula* Peters, Peters & Edmunds, 1990, and *S.milleti* Peters, Peters & Edmunds, 1990. *Simulacalarara* sp. nov. can be distinguished from all known species by distinctly elongated and narrow gills (Fig. 5A, L, M). *Simulacalanotialis* is characterised by shorter (gill IV reaches tergum VII) and wider gills that taper abruptly toward the apex (Peters et al. 1990: fig. 131). *Simulacalamassula* and *S.milleti* have gills that taper gently toward the apex, but are distinctly shorter (gill IV in middle-instar larvae reaches tergum VII–IX) and that are wider (Peters et al. 1990: figs 132, 135, respectively) than in *S.rara* sp. nov. with narrow gills and gill IV reaching or exceeding end of abdomen in middle-instar larvae. *Simulacalamassula* can be distinguished by a blackish macula on the anterior margin of the mesobasisternum and apically darkened femora (Peters et al. 1990: fig. 133) from *S.rara* sp. nov. and *S.milleti* with femora and thoracic sterna without any pattern.

##### Distribution and habitat preferences.

**Figure 7.**
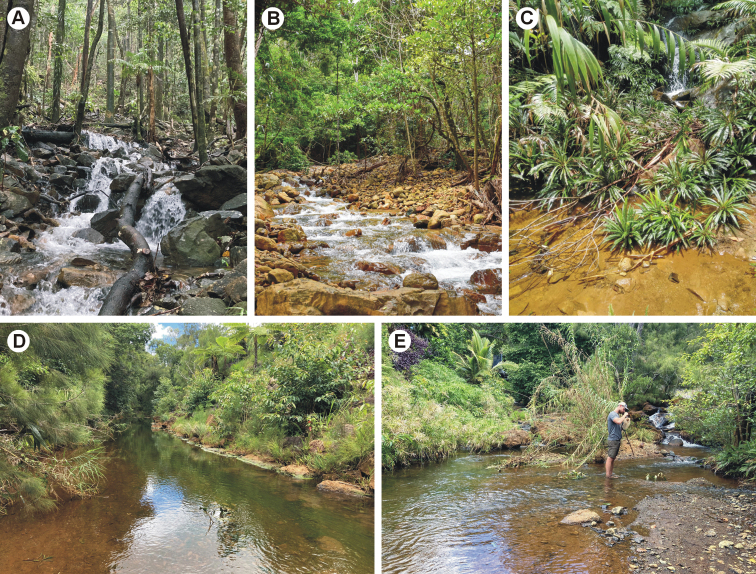
**A** type locality of *Fasciamiruspetersorum* sp. nov. **B, C** variability in habitat of *F.petersorum* sp. nov. **D, E** type locality of *Simulacalarara* sp. nov.

The species was found in the single stream Fridoline (a small stream, 3–5 m wide) in the northern province of Grande Terre (Fig. 1), on ultramafic bedrock. Its catchment includes the area of old chromite mines and active nickel laterite mines. We did not find elevated concentrations of dissolved metals (Fe, Mn, Cr, Ni) in the stream water, but magnesium content was very high (66.7 mgl^-1^) and bed surface was rich in iron precipitates. The stream was eutrophic, with total P concentration 26.2 µgl^-1^ and about 70% cover of bed substrate by filamentous green algae. The sampled section had riffles with slightly turbulent flow and gravel substrate, and predominant slow-flowing part with sandy and fine gravel substrate (Fig. 7D, E). Larvae were collected from fine gravel and sand behind stones in the riffles. They avoided similar fine-grained sediments in the slow-flowing part of the section.

## Supplementary Material

XML Treatment for Fasciamirus petersorum

XML Treatment for Simulacala rara
